# Psychosocial and Academic Implications of Food Insecurity Among International Students: A Qualitative Study in Hungary

**DOI:** 10.3390/nu17203300

**Published:** 2025-10-20

**Authors:** Soukaina Hilal, Putu Ayu Indrayathi, László Róbert Kolozsvári, Péter Torzsa, Imre Rurik

**Affiliations:** 1 Department of Family and Occupational Medicine, University of Debrecen, 4032 Debrecen, Hungary; soukaina.hilal@med.unideb.hu (S.H.); kolozsvari.laszlo@med.unideb.hu (L.R.K.); 2 Doctoral School of Health Sciences, University of Debrecen, 4032 Debrecen, Hungary; 3 Department of Public Health and Preventive Medicine, Faculty of Medicine, Udayana University, Bali 80361, Indonesia; pa_indrayathi@unud.ac.id; 4 Center for Public Health Innovation, Faculty of Medicine, Udayana University, Bali 80361, Indonesia; 5 Department of Family Medicine, Semmelweis University, 1085 Budapest, Hungary; torzsa.peter@semmelweis.hu

**Keywords:** food insecurity, international students, academics, psychosocial health, qualitative study

## Abstract

**Background/Objectives**: Recent research suggests that international students are particularly susceptible to food insecurity (FI). Yet very few studies have qualitatively examined their experiences with FI and its impacts on their health and well-being. Therefore, our study sought to address this research gap by exploring the psychosocial and academic challenges linked to FI among international students in Hungary. **Methods**: A qualitative approach using semi-structured, in-depth, one-on-one interviews was carried out from 31 July to 15 November 2024 with 15 international students studying at the University of Debrecen, Hungary. Qualitative data were analyzed using a general inductive approach with the aid of NVivo 11 software. **Results**: International students discussed five themes regarding the psychosocial implications of FI: stress and anxiety, sadness and depression, anger and frustration, guilt over financial burden, and social isolation. In terms of academic impact, four themes were mentioned: difficulty concentrating, sacrificing studies for work, diminished academic performance, and a loss of motivation and interest in their studies. **Conclusions**: This study enriches our understanding of the lived experiences of FI among international students. The findings may help shape targeted interventions that align with their needs.

## 1. Introduction

Food insecurity (FI) is generally described as “the limited or uncertain availability of nutritionally adequate and safe food or the limited or uncertain ability to acquire acceptable foods in socially acceptable ways” [1]. The global prevalence of FI has been steadily rising since the systematic collection of data began in 2014 [2]. This trend has been pushed even further by recent global challenges, such as geopolitical conflicts, the COVID-19 pandemic, and the escalating effects of climate change [3]. Recent figures from the Food and Agriculture Organization (FAO) show that roughly 2.33 billion people worldwide were affected by moderate or severe FI in 2023, a number that has remained relatively unchanged since its significant rise in 2020 during the COVID-19 pandemic [4]. In Hungary, the estimated rate of moderate or severe FI rose from 8.6% in 2019 to 10.6% in 2020, and to 15% in 2022 [5].

Students in higher educational institutions have been described as a group particularly vulnerable to FI. In a recent scoping review conducted by Nikolaus et al., the estimated prevalence of FI among college students in the United States was 41% (ranging from 10% to 75%) [6], approximately four times higher than the national prevalence among U.S. households (10.5%) [7]. In Europe, existing studies have also shown substantial prevalence rates of FI among university students [8,9,10,11,12]. For example, a recent cross-sectional study in Germany found that 33% of university students experienced FI [11], exceeding the national rate of 3.8% [13]. Another study conducted at a Spanish university reported a prevalence of 19%, with 2.5% of students experiencing moderate FI and 0.8% experiencing severe FI [12].

FI extends beyond feeling hungry; it has been linked to an array of negative health and well-being outcomes [14]. Research has demonstrated that students facing FI are more likely to have impaired sleep quality [15,16], as well as higher risks of anxiety, stress, depression [17], disordered eating behaviors [18] and chronic health problems, including type II diabetes and cardiovascular disease [14]. Furthermore, a study by Coakley et al. demonstrated that students with FI are more likely to rate their overall health as poor or fair, compared to their food-secure peers, who generally report good health [19]. FI has also been linked to reduced academic performance. As reported by Weaver et al., students with FI had significantly lower GPAs compared to those who did not experience FI [20].

International students, defined as those who travel abroad to pursue higher education, face an elevated risk of experiencing FI than their domestic counterparts [21,22]. This increased vulnerability is largely due to the unique challenges they encounter, including financial strain from costly tuition fees, limited working hours, lack of family support, and adjusting to an unfamiliar food environment [23]. A recent review published in 2021 found that international students were more likely to face FI and use food banks compared with domestic students [24]. A study by El Zein et al. found that the prevalence of food insecurity among international students at a U.S. university was 37.6%, higher than the 32.0% prevalence observed in the overall student population [25]. Furthermore, another study among Australian university students found that 61% of international students experienced FI, compared with 34% of domestic students [22]. In Europe, the issue of FI among international students has received comparatively little attention in the scholarly literature [26,27]. In Hungary, for example, the only study examining FI among international students was conducted by our research team, which found that 40.2% reported experiencing FI [26].

Despite the expanding body of research on FI among university students, there remains a notable lack of qualitative studies that capture international students’ lived experiences with FI. Understanding how international students experience FI is crucial for developing targeted strategies to address this issue within this group. Most studies conducted to date are quantitative, overlooking the rich, nuanced insights that qualitative approaches provide [24]. In addition, these studies often rely on the USDA Food Security Module, which, while useful for evaluating the prevalence and severity, does not fully capture the unique experiences and needs of international students with FI [26,28,29,30,31]. Moreover, experiences of FI can differ substantially across university contexts, and there is a scarcity of research focusing on international students in Hungarian universities, despite indications that food insecurity is common in this population [26]. Therefore, this study aimed to address this gap by using a qualitative approach to explore how FI affects international students at a Hungarian university. By focusing on personal narratives and lived experiences, this research seeks to illuminate the multifaceted impact of FI on students’ mental health, social lives, and academic performance, providing insights that quantitative measures alone cannot reveal.

## 2. Materials and Methods

### 2.1. Research Setting and Period

The current study was performed at the University of Debrecen (UniDeb) from 31 July to 15 November 2024. UniDeb is located in Debrecen, Hungary’s second-largest city, and is recognized as one of the country’s leading higher education institutions. It offers an extensive range of academic programmes across multiple disciplines. The university has a diverse student population of over 29,000, including more than 7000 international students from various countries [32].

### 2.2. Study Design

This study adopted a qualitative approach using semi-structured, in-depth, one-on-one interviews to explore international students’ experiences with FI and its effects on their psychosocial health and academics. This design was selected to allow for a flexible exploration of participants’ experiences, ensuring the collection of detailed and rich data [33].

### 2.3. Participants and Recruitment

Participants included international students who were 18 years or older, were actively enrolled in a full-time undergraduate or postgraduate study program at UniDeb, self-identified as fluent in English, and reported experiencing food insecurity. A non-probabilistic convenience sampling strategy was selected to recruit participants through social media platforms. Digital recruitment flyers outlining study details and participation instructions were distributed through the researchers’ networks. Additionally, through snowball sampling, initial participants were prompted to send the recruitment invitations to fellow students who met the study’s eligibility criteria.

Interested participants were invited to contact the primary researcher via email for further details about the study. Those who expressed interest were provided with a link to an online questionnaire, which included an information letter, an electronic consent form, and sections on demographic characteristics and food security status. Participants who matched the inclusion criteria and completed the consent form were then contacted to schedule the interview at a convenient time. Ineligible participants were sent a thank-you note acknowledging their interest and willingness to participate.

### 2.4. Materials

To determine eligibility, all students who expressed interest in the study were asked to complete the 6-item U.S. Department of Agriculture (USDA) Food Security Survey Module (FSSM), which was included in the online questionnaire. The FSSM is a self-administered questionnaire that evaluates difficulties in securing adequate food due to financial constraints. Responses indicating food access challenges, such as “yes”, “often”, “sometimes”, “almost every month”, or “some months but not every month”, were scored 1, while responses indicating no difficulties, such as “no” or “never”, were scored 0. The overall score can vary from 0 to 6, with higher scores signifying increased severity of food insecurity. Based on raw scores, food security status was classified as “high or marginal food security” (0–1), “low food security” (2–4), and “very low food security” (5–6) [34]. For this study, students with high or marginal food security were considered “food secure”, while those with low or very low food security were considered “food insecure”, consistent with the 6-item FSSM guidelines and with prior research using this measure among university students [34,35]. Only students classified as “food insecure” were eligible to participate.

In addition, demographic and socioeconomic information were collected, including, gender (female or male), age, country of origin, length of stay in Hungary (under 1 year, between 1 and 2 years, and over 2 years), study level (Undergraduate, postgraduate), sponsorship (scholarship, self-sponsor), employment (employed, not employed), and self-reported health status (good, poor).

A semi-structured interview guide was utilized to maintain focus during interviews while also allowing participants the flexibility to elaborate on their experiences. It was adapted from the one developed by Meza et al. in consultation with experts in food insecurity and public health [36]. Specifically, participants were asked to share their experiences with food insecurity since arriving in Hungary, their feelings and thoughts about it, and how it affected their psychosocial health and academics.

### 2.5. Interview Procedure

Participants were given the choice to conduct their interviews either in person or online; all opted to be interviewed online via Microsoft Teams (Microsoft Corporation, Redmond, WA, USA). Conducting interviews via online platforms offers numerous benefits, including enhanced accessibility, efficiency, cost-effectiveness, flexibility, and convenience [37]. Participants were instructed to choose a quiet, private location to avoid distractions and maintain privacy.

Before the interviews, participants were thoroughly briefed on the research purpose, the measures implemented to ensure confidentiality, the voluntary nature of their participation, and their right to opt out at any time without providing a reason. They were also informed that the interviews would be audio-recorded with their agreement and that having their cameras on was not compulsory. All participants verbally reaffirmed the written consent they had previously provided through the online questionnaire before the interview recording began.

All interviews were performed in English by the first author (S.H.), a female PhD candidate with expertise in public health and a background in nursing. The interviewer had no previous relationship with the participants. The duration of interviews varied between 12 and 44 min, with an average of 22 min. In order to protect the confidentiality of participants, numbers were used instead of their names. The interviews were performed until data saturation was achieved, at which point no additional insights were obtained. No form of compensation was provided to the study participants.

This study obtained ethical approval from the Regional Institutional Research Ethics Committee, Clinical Centre, University of Debrecen (approval number: DE RKEB/IKEB 5933-2021).

### 2.6. Analysis

A general inductive approach was deemed suitable for analyzing the data in this study because of its simplicity, efficiency, and straightforwardness. This approach facilitated the emergence of key themes from the data according to the defined evaluation objectives. The process of inductive analysis included: (1) data cleaning, (2) careful reading of text, (3) creation of categories, (4) overlapping coding and uncoded text, and (5) ongoing revision and refinement of category system [38]. All interviews that were audio-recorded were transcribed verbatim by the first author (S.H.) and reviewed by the second author (P.A.I.) to ensure accuracy. The transcribed texts were then read and coded independently by the two co-authors, who met at regular intervals to discuss and resolve any discrepancies. The final transcripts were imported into NVivo 11 (QSR International Ltd., Melbourne, Australia) to help organize themes and examine patterns across participants’ responses. All themes were further reviewed by all co-authors to ensure the validity of the results.

## 3. Results

### 3.1. Demographic and Socioeconomic Characteristics of Participants

A total of fifteen participants were interviewed, and none withdrew consent during or after their interview. The median age was 24 years (interquartile range (IQR): 22–27), with a predominance of females (60.0% vs. 40.0%). Participants originated from 11 different countries, with the largest proportion from Morocco (20.0%), followed by Nigeria and Jordan (13.0% each), and other countries (6.67% each). More than half of the interviewed participants had lived in Hungary for more than two years by the time the questionnaire was administered. Additionally, most participants were undergraduates (66.7%) and received scholarship funding (86.7%), and over half (53.3%) were unemployed. Finally, around 46% rated their health status as poor, and more than half (53.3%) reported experiencing very low food security. Detailed demographic information of the participants is displayed in Table 1.

### 3.2. Psychosocial Implications of FI

Participants discussed five themes regarding the psychosocial implications of food insecurity, including stress and anxiety, sadness and depression, social isolation, anger and frustration, and guilt over financial burden (Table 2).

#### 3.2.1. Stress and Anxiety

Stress and anxiety were common across participants’ experiences of food insecurity. Many discussed the burden of having to constantly manage their limited financial resources to ensure they had enough food. For instance, one student shared the constant stress of trying to stretch her budget to last until the end of the month:

*“The stress of making the money I have last until the end of the month so I can buy the appropriate food I need for myself is a constant stress every month. Every month, it’s the same thing”.*

(Participant 9)

Another participant described the ongoing trade-offs between meeting immediate needs and saving for future food expenses, which not only reduced his nutritional intake but also increased his stress levels:

*“You are stressed most of the time because you are always thinking, ‘I can’t buy this; I should not spend money on this because I am saving money for food later on.’ So, that is how I would say it affected my situation: more stress and less nutrition”.*

(Participant 14)

Some students also highlighted how inadequate nutrition due to food insecurity exacerbated feelings of stress and anxiety. One student, for instance, described how insufficient or poor-quality food intake triggered what he referred to as “survival anxiety”:

*“When you’re not eating enough or not eating healthy, your anxiety spikes. I don’t know if there’s research about it, but I’ve personally experienced it, and many of my friends have too. When you’re lacking food and your energy is low, you kind of have a survival anxiety. It feels like your primitive nervous system goes into stress, like a survival attack”.*

(Participant 15)

For one student, the constant stress of food insecurity was linked, in her experience, to physical health problems. She described how the ongoing worry about not having enough food affected her digestive health:

*“Every month, in the last week, I feel the same, and that’s why now I am suffering from this problem in my intestines because of this situation. Since I came here almost one year ago, I see the consequences of being under this stress and feeling like I’m not safe. You know, because I am experiencing a new feeling of not having food in my fridge. I’m not used to this. This is something new, and it has made me really stressed. Now, I’m suffering from the effects of this stress”.*

(Participant 1)

#### 3.2.2. Sadness and Depression

Many students described feeling sad and depressed as a result of their food situation, particularly due to their inability to afford foods that meet their nutritional needs and preferences. One student discussed feeling sad because of her inability to fulfill her food cravings, recalling times when her meals were extremely limited, sometimes consisting solely of plain pasta:

*“…But sometimes I feel like I really want something good. I feel sad because I want that food, but I don’t have it now. And yeah, on some days I don’t want to be dramatic because I am not that kind of person, but I think on some days I have really nothing to eat except, for example, pasta with nothing”.*

(Interview participant 1)

Another student expressed feelings of sadness and depression due to her lack of control over her food choices. She also mentioned being unable to afford foods that aligned with her cultural preferences and personal desires, such as traditional Nigerian dishes. Instead, she often had to manage with whatever limited options were available:

*“I would say I felt sad and depressed for not having a choice over what I am eating because I don’t have enough money for food. And, also, I feel like things that I want to get for myself in terms of food, I’ve not been able to get it. Sometimes I would be like, ‘Oh I want to eat a Nigerian dish.’ Because I don’t have enough money to get the food, I would just try to manage with what I have”.*

(Interview participant 3)

#### 3.2.3. Social Isolation and/or Loneliness

Some participants described how food insecurity contributed to social isolation and feelings of loneliness. For instance, one participant shared that her inability to afford sufficient food led her to withdraw from social interactions due to feelings of shame and reluctance to disclose her situation:

*“Yes, maybe because when you can’t afford food, or when you don’t have sufficient food. For me, I don’t want to stay with other people. I don’t know why. I don’t know, maybe because I feel shy to say that I don’t have too much money to buy food, or I don’t have food”.*

(Interview participant 4)

Another participant highlighted a notable shift in her social life due to food insecurity. Initially, she had a strong network of friends with whom she regularly cooked, shared meals, and exchanged traditional dishes. However, after experiencing food insecurity, she began distancing herself from her friends to avoid potential embarrassment. This withdrawal led to a loss of friendships and a deep sense of loneliness:

*“When I first came here, I did not have that problem. So, I had a lot of friends. I’d always go out with them. I make food from my country, and we all hung out, and it was all good. But once I started facing this problem, I started distancing myself from them because I did not want to go there and embarrass myself. So, I just stayed at home, like just excused myself and stayed at home, even though I wanted to hang out with friends, and now like I barely have any friends, and it feels pretty lonely”.*

(Interview participant 2)

The same participant recounted an instance in which she fabricated an excuse to avoid attending a social gathering with friends because she could not afford to contribute to the shared cost of food and felt uncomfortable disclosing her financial situation:

*“There is this one time, like my friends, they were planning to hang out and cook together. But of course, I had to buy food with them, and I did not have any money at the time. So, I just told them that I was sick. So, I did not go because I didn’t want to tell them like, hey, I don’t have money to buy food with you”.*

(Interview participant 2)

#### 3.2.4. Anger and Frustration

In many interviews, students described feeling angry and frustrated because of their food situation. One PhD student, for example, expressed anger about having to worry about basic needs like food, particularly at an advanced stage of her academic career:

*“Sometimes I feel angry. Because I feel like, at this point of my studies as a PhD student, I deserve more than thinking about how I will finish my last week here”.*

(Interview participant 1)

Another student described how returning home after long hours of studying, only to find low-quality food, led to feelings of anger:

*“When you are studying all day and you are writing articles, you are working at the laboratory for a long time, from eight maybe to six, and when you go back home, there is not good quality of food. You cannot enjoy your food because there is no money to buy more. Sure, you will be angry”.*

(Interview participant 11)

Other students expressed frustration over the high cost of food, which often limited their ability to adhere to their dietary preferences or led to skipping meals, as one student shared:

*“I feel frustrated because I wasn’t expecting the prices to be high, and I wasn’t expecting that some months I have to eat some foods that I don’t like, or I have to reduce some meals”.*

(Interview participant 4)

#### 3.2.5. Guilt over Financial Burden

Some participants expressed feelings of guilt when seeking financial support from family or friends. One student mentioned that she would occasionally call her siblings for money when unable to manage hunger, but avoided doing so frequently to prevent feeling like a burden:

*“I would say once in a while, I do call my sister or my brother. Anytime I can’t handle the hunger. I will call them for some money. But I don’t do that every time because it makes me feel like I’m bothering them. I have to take care of myself. I have to be accountable for myself”.*

(Interview participant 3)

Another student described asking her parents for additional financial support for food expenses as ‘irresponsible’, given that they were already paying her rent:

*“With my family, they don’t hesitate; as soon as I tell them something is up or I need money, they send me as much as I need or whatever they can. But I also try not to ask too much because I don’t like making them feel burdened with requests for more money, especially since I already live in a place by myself, and they’re paying the rent for it. So, for me, it seems irresponsible that on top of the rent they’re already covering, I would also have to ask them for more money to buy food”.*

(Interview participant 9)

### 3.3. Academic Implications of FI

Participants identified four themes regarding the academic implications of food insecurity, including difficulty concentrating, sacrificing studies for work, diminished academic performance, and loss of motivation and interest in studies (Table 3).

#### 3.3.1. Difficulty Concentrating

To explore the academic effects of food insecurity, students were asked, “How has your food insecurity affected your academic performance?”. Many frequently expressed that food insecurity hindered their ability to concentrate when studying. Students described how hunger disrupted their focus, thereby affecting their learning:

*“There are times when the limited resources also affect how I cope with learning. Because, for example, if in the afternoon I eat bread and have a class until six o’clock, then around three o’clock, I’m already hungry. I become unfocused”.*

(Interview participant 6)

*“When I am hungry, I can’t read. I can’t read anything; nothing will be entering my head. Whenever I open the book, all I see is food. So, if I have enough money to eat when I’m hungry, I’ll be able to concentrate on my studies”.*

(Interview participant 3)

Beyond the physical effects of hunger, students also highlighted how the stress surrounding food insecurity distracted them from their academics. One participant described his inability to focus on his studies when his mind was constantly preoccupied with worries about food:

*“If I stress too much about my food, it actually occupies my mind. I couldn’t really focus on what the teacher is saying”.*

(Interview participant 6)

#### 3.3.2. Sacrificing Studies for Work

Another recurring theme among students was the challenge of balancing paid work with their studies. Many students reported that taking on part-time jobs to manage food insecurity and other expenses negatively impacted their academic progress. One student explained:

*“I tried to work part-time to cover these expenses. So, this really affects my academic progress because I was losing time working instead of studying”.*

(Interview participant 4)

Another student noted that during the last exam period, he wanted to study more but was unable to complete a course due to working simultaneously. He emphasized that even working four to five hours a day consumes valuable time that could otherwise be dedicated to covering a significant portion of the course material:

*“Many times. Even during the last exam period, I wanted to study more, but I couldn’t complete one course because I was working at the same time. Even if you’re working four or five hours a day, that’s a lot of time. In that time, you could cover a lot of your syllabus”.*

(Interview participant 15)

#### 3.3.3. Diminished Academic Performance

Many students felt that FI had a negative impact on their academic performance. One participant described how not having enough money for food left her feeling stressed and unable to concentrate while preparing for an exam. She explained that because she wasn’t well-fed, her brain was “barely working”, leading to poor results:

*“Last semester I had this exam, and it was like around the end of the month. I barely had any money or food at home. So, I was not eating very well. And I was like stressed because of the exams and also stressed about the fact that I don’t have enough money or food at home. So, when I was preparing for the exam, my brain was barely working because, like, um, there was so much stress, and I’m not well fed. And so, you know, my results were not very good on the exam, unfortunately”.*

(Interview participant 2)

Another student shared a similar experience, recalling how skipping meals before an exam led to feelings of dizziness and anxiety, which hindered her ability to concentrate. As a result, she did not perform well and had to retake the exam:

*“I was able to read, I studied for the exam, and did not eat the night before the exam. Before going for the exam, I did not eat. So, going to the exam and seeing the paper, my heart started pounding. I started feeling dizzy, but I was able to finish the exam. But I know I couldn’t score an excellent grade, so I had to go back for the retake”.*

(Interview participant 3)

#### 3.3.4. Loss of Motivation and Interest in Studies

In some interviews, students reported losing motivation and interest in their studies due to their food and financial situations. One student described feeling demotivated and frustrated after struggling to concentrate due to hunger:

*“I felt mad at myself for putting myself in this situation for not feeding myself enough. And also it felt demotivating because I feel like I’m doing too much effort. But like, then I got a bad grade because I could not concentrate enough”.*

(Interview participant 2)

Another student shared that her year was unsuccessful and that she had lost motivation for her degree. She even sought help from a university-provided psychiatrist to discuss her uncertainty about continuing her studies:

*“So, it wasn’t a successful year for me, but I will tell you how it’s affecting me that I have no more motivation for this degree. And I am like, I even went to a psychiatrist to talk with her—that the university offer—because I was not sure if I want this anymore. This degree brings a lot of problems with it, just because of the lack of money. And yeah, I would say that it’s affecting my motivation”.*

(Interview participant 1)

## 4. Discussion

This study is, to our knowledge, the first to qualitatively explore the lived experiences of international students with food insecurity in Hungary and how it affects their psychosocial health and academics. International students facing food insecurity described a range of psychosocial challenges, including stress and anxiety, sadness and depression, anger and frustration, guilt over financial burdens, and social isolation. They also felt that food insecurity negatively affected their academic lives, leading to reduced concentration, sacrificing study time for work, diminished performance, and a loss of motivation and interest in their studies. Our findings extend earlier quantitative research by offering context and explanations to the observed associations between food insecurity, psychosocial well-being, and academic outcomes among international students. Insights from this study may help identify areas for interventions and support programs that alleviate food insecurity, thereby promoting both mental health and academic success among this overlooked population.

To date, few qualitative studies have examined the problem of FI among international students, with very limited attention given to its implications for their well-being and academic performance. In 2021, Hanbazaza et al. conducted a qualitative descriptive research study at the University of Alberta, Canada, using semi-structured interviews with a purposive sample of 11 international students who had accessed an on-campus food bank. While their study employed different methodologies, several themes from our research align with their findings, including stress, anxiety, anger, social withdrawal, difficulty concentrating, and poor academic performance [39]. The consistency of these results across different educational settings reinforces the substantial effects of FI on international students’ psychosocial health and academics.

Our findings corroborate existing evidence on the association between FI and psychosocial health among the general population, including university students [40,41,42,43,44]. For instance, a large-scale study revealed that students who reported being food insecure had significantly higher odds of anxiety, depression, loneliness, and even self-injurious behaviours, relative to those who did not report FI [43]. Similarly, another study involving both local and international students at a university in Australia found that those facing FI were more likely to report poor mental health and well-being [28]. In a qualitative study by Lemp et al., conducted at a large Midwestern university in the United States, students described how FI led to a range of emotional and mental health difficulties, including anxiety, stress, depression, frustration, guilt, shame, and embarrassment [45]. As suggested by Bruening et al., the relationship between FI and emotional health may be bidirectional, wherein FI may contribute to compromised emotional health, and compromised emotional health may increase the likelihood of experiencing FI [41]. Although this study investigated the emotional effects of FI, it could be that pre-existing mental health challenges impaired some international students’ ability to maintain part-time jobs or navigate financial difficulties, thereby contributing to their experiences of food insecurity. Therefore, additional research is needed to better understand the underlying mechanisms and clarify the direction of causality.

The impact of FI on academics has been well-documented in previous studies among university students and children [20,46,47,48,49]. In Lemp’s study, food-insecure students reported difficulty concentrating, diminished mental capacities, reduced academic performance, less motivation to study, prioritizing work over attending class, and even reconsidering their decision to pursue graduate school [43]. Hagedorn et al. found significant differences in GPA between food-secure and food-insecure students, with food-secure students having an average GPA of 3.51, compared to 3.33 for their food-insecure peers [50]. Another study conducted at a Minority Institution in the southwestern United States revealed that, even when controlling for other factors, students with FI had a significantly higher likelihood of failing, withdrawing from courses, or leaving school entirely [47]. Research also suggests that psychosocial factors, including depression, anxiety, and stress, mediate the relationship between FI and academic performance. For instance, Raskind et al. (2019) revealed that FI contributed to psychological distress, which in turn negatively affected GPA and academic success [44]. These findings indicate that addressing FI requires a holistic approach that incorporates mental health support and other psychosocial interventions to mitigate its effects on student achievement. Students, as part of the general population, are facing with recent challenges regarding changing knowledge on proper nutrition [51].

The findings of this study have several important practical implications. International students’ narratives suggest that food insecurity extends beyond limitations in dietary quality and is associated with challenges to well-being, academic engagement, and social integration. These experiences highlight the need for universities to develop and implement strategies that prevent and address FI among international students and mitigate its effects on their overall well-being. Programs such as campus food pantries, food scholarships, and meal swipe plans are effective approaches to support students in this context [52]. In addition, educational initiatives such as offering online courses, workshops, or cooking sessions that focus on preparing healthy and budget-friendly meals can also help students make informed financial and dietary decisions [53]. These initiatives should be integrated with accessible mental health and counselling services, peer support groups, and mentorship programs. International students at the University of Debrecen have access to free psychological counseling services delivered by qualified psychologists. Promoting awareness of these services is essential for supporting students’ mental health and ensuring they can effectively utilize available resources.

Broader societal and policy measures are also necessary, as interventions at the university level alone cannot fully tackle the underlying structural causes of FI among international students. While strategies such as campus food pantries, meal vouchers, and emergency financial support provide important short-term relief, these measures do not address the root economic and social factors leading to FI. Sustained improvements require comprehensive policies that reduce the overall cost of living for international students, including affordable housing, transportation, and healthcare, as well as programs that enhance access to affordable, nutritious, and culturally relevant foods [54].

The present study has several noteworthy strengths that enhance the credibility and depth of its results. First, the application of a general inductive approach allowed for a thorough and nuanced examination of the research topic. Second, qualitative one-on-one, in-depth interviews with open-ended questions facilitated the collection of rich and detailed insights into international students’ experiences with food insecurity. Third, the interviewer’s shared background as an international student in Hungary helped build a strong rapport with participants, encouraging them to speak openly about their experiences. Fourth, we believe that interviews were conducted until data saturation was attained, as no additional themes emerged in the final interviews. Finally, the use of NVivo software facilitated the organization and analysis of the data.

Notwithstanding the noteworthy insights offered by this study, certain limitations need to be considered. First, this study was conducted at a single university in Hungary, so the experiences captured may not be generalizable to international students at other institutions. Second, the use of convenience and snowball sampling, while effective for reaching international students willing to share their experiences with food insecurity, may have introduced selection bias. Additionally, all in-depth interviews were carried out online using audio communication only. While this method offers convenience, it limits the ability to capture nonverbal cues that could provide additional context to participants’ responses.

Despite these limitations, the study targets a key gap in existing research by focusing specifically on international students. Our findings offer foundational insight into how food insecurity influences the psychosocial health and academic performance of international students and can guide future research aimed at identifying effective university-based interventions to mitigate food insecurity and promote the well-being of this vulnerable population. Building on this work, future studies should examine the long-term effects of FI on international students’ mental health and overall well-being, using both qualitative and quantitative approaches to develop a more comprehensive understanding of this issue.

## 5. Conclusions

This qualitative study investigated the lived experiences of international students with FI at a Hungarian university and its impact on their psychosocial health and academics. Participants expressed that FI had significant effects on their mental health, social lives, and academic performance. Specifically, many described experiencing stress and anxiety, sadness and depression, anger and frustration, and guilt over financial burden. Some participants also spoke of feeling ashamed or embarrassed due to FI, leading them to withdraw from social interactions, which in their experience resulted in profound loneliness and social isolation. In terms of academics, participants reported that FI impaired their concentration, reduced motivation, limited study time, and ultimately undermined their academic performance. These findings suggest that FI among international students cannot be viewed as an isolated issue but as a multifaceted challenge that affects many aspects of their daily lives and well-being. They also point to the urgent need for targeted interventions and strategies to mitigate FI and support the overall well-being of this student population.

## Figures and Tables

**Table 1:** Demographic and socioeconomic characteristics.

Characteristics		*n* (%)
Age	Median (IQR)	24.0 (22–27)
Age (years)	21–25	10 (66.7%)
	26–30	3 (20.0%)
	31–36	2 (13.3%)
Gender	Female	9 (60.0%)
	Male	6 (40.0%)
Country of origin	Morocco	3 (20.0%)
	Nigeria	2 (13.3%)
	Jordan	2 (13.3%)
	Tunisia	1 (6.67%)
	Kenya	1 (6.67%)
	Syria	1 (6.67%)
	Iraq	1 (6.67%)
	Cambodia	1 (6.67%)
	Philippines	1 (6.67%)
	Pakistan	1 (6.67%)
	India	1 (6.67%)
Years in Hungary	Less than 1 year	2 (13.3%)
	Between 1 and 2 years	5 (33.3%)
	More than 2 years	8 (53.3%)
Study level	Undergraduate	10 (66.7%)
	Postgraduate	5 (33.4%)
Employment	Scholarship	13 (86.7%)
	Self-sponsor	2 (13.3%)
Self-Reported Health	Good	8 (53.3%)
	Poor	7 (46.7%)
Food Security Status	Low food security	7 (46.7%)
	Very low food security	8 (53.3%)

**Table 2:** Emerging themes and illustrative quotes related to the psychosocial implications of FI.

Themes	Illustrative Quotes
Stress and anxiety	*“Sometimes when you feel hungry, there’ll be a lot of emotions running through your head, a lot of things… I was in that situation that sometimes, if my parents or my mom called me, after the call, because I don’t want them to see me in tears, I would start crying, like, oh my God, I want to go back home. I felt stressed and anxious at that point”.* (Interview participant 3) *“You are stressed most of the time because you are always thinking, ‘I can’t buy this; I should not spend money on this because I am saving money for food later on.’ So, that is how I would say it affected my situation: more stress and less nutrition”.* (Interview participant 14)
Sadness and depression	*“Yeah, as I have told you, sometimes I need balanced food. But because the money I have is so little, I cannot manage to buy that. So actually, this makes me depressed”. *(Interview participant 7) *“…But sometimes I feel like I really want something good. I feel sad because I want that food, but I don’t have it now. And yeah, on some days I don’t want to be dramatic because I am not that kind of person, but I think on some days I have really nothing to eat except, for example, pasta with nothing”.* (Interview participant 1)
Social isolation and/or loneliness	*“There is this one time, like my friends, they were planning to hang out and cook together. But of course, I had to buy food with them, and I did not have any money at the time. So, I just told them that I was sick. So, I did not go because I didn’t want to tell them like, hey, I don’t have money to buy food with you”. *(Interview participant 2) *“Yes, maybe because when you can’t afford food, or when you don’t have sufficient food. For me, I don’t want to stay with other people. I don’t know why. I don’t know, maybe because I feel shy to say that I don’t have too much money to buy food, or I don’t have food”.* (Interview participant 4)
Anger and frustration	*“I feel a little bit angry in a way. Like maybe if I did not choose to come here, maybe I would have been like home with my family and have enough food”.* (Interview participant 2) *“I feel frustrated because I wasn’t expecting the prices to be high, and I wasn’t expecting that some months I have to eat some foods that I don’t like, or I have to reduce some meals”.* (Interview participant 4)
Guilt over financial burden	*“I rely heavily on my parents because I couldn’t find a job yet… Depending on them, even now as an adult, makes me feel guilty”.* (Interview participant 8) *“I would say once in a while, I do call my sister or my brother. Anytime I can’t handle the hunger. I will call them for some money. But I don’t do that every time because it makes me feel like I’m bothering them. I have to take care of myself. I have to be accountable for myself”.* (Interview participant 3)

**Table 3:** Emerging themes and illustrative quotes related to the academic implications of FI.

Themes	Illustrative Quotes
Difficulty concentrating	*“It lowers my concentration… Like this morning, I went to the office at around eight, but later, I was leaving the office because my concentration was already gone. I only had breakfast, and I don’t have anything to eat now. So my concentration just went down”.* (Interview participant 15) *“When I am hungry, I can’t read. I can’t read anything; nothing will be entering my head. Whenever I open the book, all I see is food. So, if I have enough money to eat when I’m hungry, I’ll be able to concentrate on my studies”.* (Interview participant 3)
Sacrificing studies for work	*“When I got my job, there was a time, it was the exam period, and I was just shocked-oh, tomorrow is the deadline. I didn’t have the time to do it. And I was just shocked, because I saw how having a job affected also my studies… And even if it’s a four-hour or six-hour job in one day, it’s actually tiring. It drains your energy, so at the end of the day, you just wanted to rest. You couldn’t study; that’s how it affected my studies”. *(Interview participant 7) *“I tried to work part-time to cover these expenses. So, this really affects my academic progress because I was losing time working instead of studying”.* (Interview participant 4)
Diminished academic performance	*“I was able to read, I studied for the exam, and did not eat the night before the exam. Before going for the exam, I did not eat. So, going to the exam and seeing the paper, my heart started pounding. I started feeling dizzy, but I was able to finish the exam. But I know I couldn’t score an excellent grade, so I had to go back for the retake”. *(Interview participant 3) *“Last semester I had this exam, and it was like around the end of the month. I barely had any money or food at home. So, I was not eating very well. And I was like stressed because of the exams and also stressed about the fact that I don’t have enough money or food at home. So, when I was preparing for the exam, my brain was barely working because, like, um, there was so much stress, and I’m not well fed. And so, you know, my results were not very good on the exam, unfortunately”.* (Interview participant 2)
Loss of motivation and interest in studies	*“I could just go back home… I’m just suffering. I felt like giving up on these studies and going back home”. *(Interview participant 7) *“So, it wasn’t a successful year for me, but I will tell you how it’s affecting me that I have no more motivation for this degree. And I am like, I even went to a psychiatrist to talk with her—that the university offer—because I was not sure if I want this anymore. This degree brings a lot of problems with it, just because of the lack of money. And yeah, I would say that it’s affecting my motivation”.* (Interview participant 1)

## Data Availability

The transcribed interviews, anonymized, are available from the corresponding author upon reasonable request. The data are not publicly available due to ethical considerations related to participants’ confidentiality and privacy.
